# Disability as an Interpersonal Experience: A Systematic Review on Dyadic Challenges and Dyadic Coping When One Partner Has a Chronic Physical or Sensory Impairment

**DOI:** 10.3389/fpsyg.2021.624609

**Published:** 2021-03-01

**Authors:** Isabella C. Bertschi, Fabienne Meier, Guy Bodenmann

**Affiliations:** Clinical Psychology for Children/Adolescents and Couples/Families, Department of Psychology, University of Zurich, Zurich, Switzerland

**Keywords:** health impairments, dyadic coping, chronic illness, interdependence, couples, disability, dyadic challenges, mutual sharing

## Abstract

Chronically disabling health impairments affect an increasing number of people worldwide. In close relationships, disability is an interpersonal experience. Psychological distress is thus common in patients as well as their spouses. Dyadic coping can alleviate stress and promote adjustment in couples who face disabling health impairments. Much research has focused on dyadic coping with cancer. However, other health problems such as physical and sensory impairments are also common and may strongly impact couple relationships. In order to promote couples' optimal adjustment to impaired health, the identification of disability-related relationship challenges is required. Furthermore, ways in which dyadic coping with these challenges may benefit couples could inform researchers and practitioners how to support couples in coping with health impairments. Accordingly, the aims of this study were to systematically review dyadic challenges and dyadic coping when one partner has a chronically disabling physical or sensory impairment. Out of 873 articles identified through database searches, 36 studies met inclusion criteria. The disability-related dyadic challenges identified in the review were changed roles and responsibilities within the couple, altered communication, compromised sexual intimacy, and reduced social participation. These challenges were reported to burden both partners and the couple relationship. Dyadic adjustment benefitted from a we-perspective, i.e., when couples viewed the disability as a shared challenge and engaged in conjoint dyadic coping. The results suggest that patient/care recipient and partner/caregiver roles should be de-emphasized and that disability should be recognized as an interpersonal experience.

## Introduction

Over a billion people worldwide are estimated to live with some form of disability. The most important causes of disability are impairments associated with chronic health conditions, e.g., visual impairment as a secondary consequence of diabetes. Due to population aging, chronic health conditions and associated disability are expected to steadily increase in the future (World Health Organization, 2015). In the European Union, for instance, 1 in 10 adult citizens reports severe physical or sensory disability (Eurostat, 2020) and almost two thirds of all adults are married or cohabiting (Corselli-Nordblad and Gereoffy, 2018). With more people living with disability, more relationship systems will be impacted by the consequences of disability. The aim of this study is thus to systematically review the challenges couples face when one partner has a chronically disabling physical or sensory impairment and what is known about dyadic coping in this context.

### Psychosocial Consequences of Disability in Patients and Spouses

Chronic health impairments and disability can cause significant psychological distress for patients. For instance, symptoms of depression or pronounced anxiety are common across a seemingly diverse range of conditions including multiple sclerosis (Dennison et al., 2010), spinal cord injury (Le and Dorstyn, 2016), or vision loss (Nyman et al., 2012). Although initially intense emotional reactions to symptom onset and diagnosis may be followed by more moderate distress, the chronicity of impairments and the often progressive course of the underlying health condition urge the patient to permanently adjust to living with the impairments and their consequences (e.g., in multiple sclerosis; Desborough et al., 2020). Adjustment to a “new normal” requires patients with chronic health conditions to cope with disabling health impairments on a daily basis, for example, by following a treatment regimen, managing the financial impact of treatments, or changing leisure time activities and social interactions to accommodate the impairment (Stanton et al., 2007; Badr and Acitelli, 2017).

Disability is an interpersonal experience as it also affects the people patients are close with. Close relationship partners exhibit similar levels of psychological distress as patients. For instance, meta-analytic evidence suggests that patient and spouse psychological distress are comparable, i.e., distress does not significantly differ between patients and partners. Both partners, however, exhibit higher prevalence of depression and anxiety compared with healthy controls (Hodges et al., 2005; Mitchell et al., 2013). While other family members also suffer, romantic partners are particularly prone to experiencing distress related to the patient's impairment. Firstly, spouses are strongly affected because the impairment and its consequences represent a threat to the health and well-being of someone close to them and to the life they have built together. For instance, partners of breast cancer patients often exhibit harm/loss appraisals and intrusive thoughts in relation to cancer, indicating intense preoccupation related to their partner's health condition (Steiner et al., 2014). Secondly, cohabiting partners often take over caregiving tasks and help the patient manage treatment regimens. This provision of practical support to the patient can be experienced as distressing (Adelman et al., 2014). Thirdly, spouses are generally the patients' main confidant (Collins and Feeney, 2000). Being empathetic to the patient's distress, partners may experience contagion with (negative) emotions (Coyne et al., 1987; Bodenmann, 1995; Revenson et al., 2016). Also, as patients confide in them, romantic partners are often the primary source of emotional support for patients (Revenson, 1994). However, the expectation that they should provide emotional support may cause additional distress for partners.

These pathways show how a stressor pertaining originally to the patient, the health impairment, can come to affect the patient's romantic partner as well. The pathways are consistent with the Systemic Transactional Model (STM; Bodenmann, 1995, 1997, 2005) of stress and coping. The STM details how within a committed romantic relationship, certain situations can cause stress beyond the person originally faced with the situation and how, therefore, stress may affect the couple as a unit. The joint affectedness of both members of the couple suggests that chronically disabling health impairments ought to be conceptualized as “we-stress.”

### We-Stress and Conjoint Forms of Dyadic Coping

We-stress describes any stress directly concerning the couple as a unit, e.g., the birth of a child or financial hardship (Bodenmann, 1995; Bodenmann et al., 2016). In the context of serious illness, the term “we-disease” has been suggested (Kayser et al., 2007). Both terms underline that couples face severe life stressors such as chronic disease of one partner as shared interpersonal experiences (Leuchtmann and Bodenmann, 2017). Chronically disabling health impairments match the criteria of we-stress well because their consequences clearly affect both partners, as outlined above, and they require permanent (re)adjustments in the couples' everyday lives.

However, we-stress not only implies the shared experience of stress within the couple, but it also suggests that the couple holds shared resources to counteract their stress. One such resource is dyadic coping. Dyadic coping encompasses supportive actions from one partner to the other as well as conjoint coping efforts of both partners (Bodenmann, 1995). In community samples, dyadic coping was found to be beneficial for individual (e.g., Gabriel et al., 2016) and dyadic adjustment (e.g., Falconier et al., 2013; Randall et al., 2016). However, common dyadic coping (CDC), a conjoint form of dyadic coping, is most suitable in response to we-stress. In CDC, both partners are involved in coping with stress that affects them both. Symmetrical engagement in CDC not only lowers stress in both partners, but it also strengthens their mutual identification as a unit, i.e., their sense of we-ness (Bodenmann, 2005). Meta-analytic evidence suggests that conjoint forms of dyadic coping such as CDC are the strongest predictor of relationship satisfaction in community samples when compared with other forms of dyadic coping (Falconier et al., 2015). In the context of chronic health impairments, systematic reviews similarly show that conjoint dyadic coping is consistently associated with good relationship functioning (Traa et al., 2015). In couples coping with the wife's breast cancer, higher CDC was associated with higher relationship quality and fewer depressive symptoms in both patients and partners (Rottmann et al., 2015) and with lower psychological distress in partners (Meier et al., 2019). In couples coping with diabetes, CDC was related to patients' adherence to dietary and exercise regimens which is vital to avoid serious complications (Johnson et al., 2013). This further underlines the close links between relational and individual health. Another form of conjoint dyadic coping is communal coping. Communal coping refers to collaborative efforts to cope with a shared stressor that affects more than one individual (Lyons et al., 1998), i.e., we-stress. In couples facing type 2 diabetes, communal coping was related to better relationship quality perceived by the patient and reduced patient and partner distress (Helgeson et al., 2017), further supporting the relevance of conjoint forms of dyadic coping in adjusting to health impairments.

### Dyadic Coping Across Health Impairments

Although dyadic stress and coping frameworks have received growing interest in the context of chronic health impairments, the range of health conditions that have received scholarly attention is relatively narrow. Analyzing included studies in a comprehensive review of couples' coping with chronic illness (Berg and Upchurch, 2007), cancer populations are by far the most studied, particularly breast cancer patients and their spouses (e.g., Feldman and Broussard, 2006). Other, much less frequently studied conditions included cardiovascular diseases (e.g., Coyne and Smith, 1991) and arthritis (e.g., Keefe et al., 1999). A recent systematic review on dyadic coping showed a similar picture indicating that the health conditions dyadic coping research focuses on have not changed much over the last decades (Falconier and Kuhn, 2019). A bias toward cancer research in the literature may be explained by funding priorities due to high prevalence and mortality rates of cancer. However, knowledge on dyadic coping in the context of a broader range of health impairments is needed. For example, multiple sclerosis is among the most important causes of disability among young adults in their child-rearing years (Kingwell et al., 2013). As such, it poses specific dyadic coping challenges for couples as do other health conditions. More knowledge is therefore needed, for instance, to develop targeted interventions for couples coping with multiple sclerosis and other chronically disabling impairments. Discerning what stressors and mechanisms in dyadic coping are comparable across impairments and what might be specificities of others requires a better understanding of factors like the duration and intensity of the stress experience caused by the impairment (Randall and Bodenmann, 2009) and other contextual factors such as controllability and predictability of symptoms (for an overview of contextual factors, see, e.g., Berg and Upchurch, 2007). For instance, in the context of cancer, couples may face a highly stressful acute illness phase which is usually followed by a remission phase with decreasing stress levels in the best case or lethal development in the worst case. Accordingly, changes in quality of life of cancer patients and their partners appear to be a function of the phase of illness/survivorship and stressors associated with the respective phase (Song et al., 2011). Integrating evidence on health conditions with differing contextual characteristics that pose unique challenges for couples may therefore productively expand research on stress and dyadic coping in the context of health.

Although some relationship challenges such as heightened uncertainty about the future may be comparable across many chronic health conditions (e.g., Rolland, 1994), there may be specific dyadic challenges and relationship strains due to impairments that lead to irreversible physical and/or sensory disability. Their potential relationship impact is very high due to the interference of physical or sensory impairments with key domains of romantic relationships such as sexual function or couple communication. For example, erectile dysfunction has been reported in up to 80% of males with spinal cord injury (Jia et al., 2016). In couples where one partner has multiple sclerosis, fatigue and fear of pain are often reported as barriers to satisfying sexual relationships (Marck et al., 2016). Evidence also suggests low levels of sexual activity in couples where one partner had acquired deafblindness (Lehane et al., 2017b). Sensory dysfunction might interfere with dyadic communication, e.g., reducing the patient's ability to perceive subtle visual or auditory cues of sexual interest in their partner. This, in turn, could lead to reduction in sexual activity. Dyadic communication can also be altered when the couple is faced with physical impairments that affect verbal and/or non-verbal expression. For example, problems with motor speech production are common in Parkinson's disease and frequently cause communication breakdown in dyads (Altaher et al., 2020). Furthermore, physical and sensory disability impact the mobility and independence of the affected individual. Mobility restrictions may challenge couples' established division of responsibilities. For example, couples may need to find new ways to distribute household tasks or leisure time (e.g., Hodgson et al., 2004). These examples show the interpersonal relevance of physical and sensory disability as they can lead to significant dyadic challenges in affected couples (i.e., we-stress). Previous reviews have established associations between physical and sensory disability and individual psychological well-being in couples (e.g., Ennis et al., 2013; Lehane et al., 2017a), supporting the notion that physical and sensory disability cause we-stress. However, to our knowledge, the specific dyadic challenges that cause such we-stress and how couples dyadically cope with chronically disabling health impairments have not been systematically reviewed yet. We will thus address the examination of dyadic challenges and dyadic coping related to physical and sensory disability in the current study.

### Need for Integration of Quantitative and Qualitative Evidence

Another concern in the field of dyadic coping with chronic health impairments that we aim to address is the lack of integration of quantitative and qualitative evidence. Empirical work based on traditional dyadic coping frameworks such as the STM has mainly relied on quantitative data using validated scales to measure the related constructs (e.g., Dyadic Coping Inventory; Bodenmann, 2008). Qualitative evidence on how couples adjust to chronic health impairments has steadily increased in parallel. Yet, few qualitative studies have made explicit reference to dyadic coping frameworks. This parallel development of quantitative and qualitative research poses the risk that findings are not sufficiently integrated for the development of theory and interventions for couples coping with chronically impaired health. One exception is the study of Kayser et al. (2007) that integrated qualitative analyses of couple interviews with existing dyadic coping theory. They identified key aspects of coping with breast cancer as a couple including appraisals of cancer as we-stress. The term we-disease was deduced from this qualitative study and has thereby, in turn, enriched quantitative research. The study further underlined the importance of reciprocal communication to identify each partner's emotional response to the situation and their coping needs and coordination of individual and joint coping responses. Sallay et al. (2019) also used qualitative methodology to study dyadic coping in the context of chronic health impairments. Their interviews with family members of chronically ill individuals revealed how dyadic coping in the families was shaped by the sociophysical environment, e.g., how spatial arrangements were used to communicate stress and how they contributed to coping by creating distance or closeness. Both examples highlight the important insight qualitative evidence can add to research on couples who are coping with chronically impaired health and how the integration of quantitative and qualitative evidence contributes to stimulating future research.

### The Present Study

Chronically disabling health impairments will become more frequent as populations worldwide age. More and more couple relationships are urged to cope with the dyadic challenges and stress that are caused by one partner's disability. Irreversible physical and sensory disability in particular can have a significant impact on romantic relationships but have, however, not been a major focus of dyadic coping research so far. Furthermore, existing quantitative and qualitative research on couples coping with chronically impaired health have been poorly integrated despite innovative potential of such integration. Consequently, the present study aims to (1) systematically review dyadic challenges in couples coping with chronically disabling physical and sensory impairments and to (2) synthesize existing research on dyadic coping in these couples.

## Methods

The systematic review followed the recommendations in the Preferred Reporting Items for Systematic Reviews and Meta-Analyses (PRISMA) statement (Liberati et al., 2009) and the subsequent PRISMA-P 2015 checklist for review protocols (Moher et al., 2015).

### Search Strategy

The literature search was conducted in July 2020 using the databases APA PsycINFO, CINAHL, Medline, and PSYNDEX accessed *via* EBSCOhost. The combination of databases allowed for diversity in disciplinary backgrounds of studies given that research on interpersonal relations in the context of health impairments lies at the intersection of different fields, e.g., social psychology, rehabilitation nursing, and communication sciences. All searches were limited to research published after 1990 when dyadic coping frameworks had started to emerge. For the purpose of deriving search terms concerning health impairments, sensory impairments were defined as functional losses of sight or hearing. Physical impairments were defined as limitations on a person's physical functioning and mobility that are primarily rooted in functional changes to the neuromusculoskeletal system. Chronic illnesses that may result in limitations of patients' physical functioning and mobility as a secondary consequence of the primary diagnosis were excluded to clearly delineate the scope of this review from previous reviews on dyadic coping with chronic illness in general (e.g., Berg and Upchurch, 2007). Health conditions causing such impairments were identified and the respective keywords derived. The search terms for the first aim on dyadic challenges consisted of a combination of (1) types of health conditions or impairments, as defined above; (2) keywords for a dyadic/couple/relationship focus; and (3) keywords for dyadic adjustment. An example search string to be found in the title or abstract of a study was as follows: (“spinal cord injury” or paraplegia or tetraplegia or hemiparesis or hemiplegia or “traumatic brain injury” or “multiple sclerosis” or arthritis or parkinson^*^ or stroke) AND (couple^*^ or dyad^*^ or spous^*^ or “significant other^*^” or wife or wives or husband^*^ or marital or married or marriage or “committed relationship^*^”) AND (adjustment or “relationship satisfaction” or “relationship quality”). The search terms for reviewing literature for the second aim on dyadic coping with chronically disabling physical and sensory impairments consisted of a combination of (1) the types of health conditions or impairments relevant to this review and (2) keywords for dyadic coping. An example search string to be found in either the title or abstract of a study was as follows: (“sensory loss” or “sensory impairment” or “sensory dysfunction” or “vision loss” or “visual impairment” or “visually impaired” or “vision impairment” or “low vision” or blind^*^ or “hearing loss” or “hearing impairment” or “hearing impaired” or “hard of hearing” or deaf^*^ or “dual sensory loss” or “vision disorder^*^” or “eye disorder^*^” or “hearing disorder^*^”) AND (“dyadic coping” or “communal coping” or “collaborative coping” or “coping congruence” or “cooperative coping” or “couple coping” or “relationship-focused coping” or “spousal support” or “partner social support”). Complete search strings can be found in the Supplementary Material. Additional articles were subsequently identified through hand searches and by inspecting the reference lists of articles identified by the original search queries and review articles on dyadic coping (Berg and Upchurch, 2007; Falconier and Kuhn, 2019).

### Study Selection

Inclusion criteria for the review covered the following: (a) peer-reviewed journal article reporting on an empirical study or a systematic review, (b) full text available in English, (c) sample consisting of persons with an acquired chronic or progressively worsening physical or sensory impairment (see search terms) or their romantic partners, and (d) focus on dyadic processes or dyadic outcomes, e.g., relationship satisfaction, sexual intimacy, and dyadic coping. The inclusion criteria were chosen to identify studies with a genuinely dyadic perspective on the implications of the selected health impairments for the couple relationship. Exclusion criteria were (a) study protocol or psychometric article, (b) non-progressive congenital impairment, (c) focus on individual processes or outcomes only, and (d) dyadic variable studied solely as predictor of individual outcomes. Thus, we wanted to ensure that genuinely dyadic studies were included as opposed to studies that only considered dyadic variables in one individual of the couple and studied its association with purely individual outcomes, e.g., depressive symptoms. Lastly, we also excluded studies if (e) their results were not discernible for romantic partners and other family caregivers. Studies in which all or part of the results were clearly identifiable as pertaining to romantic partners were retained. No restrictions on study type were made to ensure that evidence from qualitative and quantitative research would be considered.

Based on the above criteria, titles and abstracts of the database-identified articles were screened for relevancy by the first author (ICB) and three independent screeners. In case of insufficient information from the title and abstract, full texts were retrieved and screened for relevancy. Random double-checks of screening decisions were conducted to ensure the quality of the screening process. Following the initial title and abstract screening process, the first author and two screeners checked the full articles for eligibility. Information on study sample, study design, and phenomena of interest or study variables were inserted into a database. Screeners noted their decision on inclusion or exclusion for every full text. In case of doubts, the respective full text was discussed among the screening team until consensus was reached.

### Data Extraction

After title and abstract screening, the first author and two data extractors completed information in the database for all included articles and randomly double-checked entries. Data extraction included authors, year of publication, setting, sample, study design and methods, investigated variables or phenomena, findings, and conclusions. Quality assessments were added for quantitative and qualitative studies separately. Quality assessment of quantitative studies relied on an adapted version of the Quality Assessment Tool for Observational Cohort and Cross-Sectional Studies proposed by the National Institute of Health (2020). Of the tool's 14 original questions, three were dropped because they were only applicable to cohort studies. Eleven questions were retained covering the following quality criteria: clear statement of research question or objective, sufficient description of study population, appropriateness and uniform application of inclusion and exclusion criteria for participants, provision of sample size justification, measurement of independent variables (IV) prior to measurement of dependent variables (DV), sufficient time frame between measurements of IV and DV, variation in IV, good validity and reliability of IV, multiple assessment of IV, good validity and reliability of DV, and measurement and statistical control of confounders. Assessors answered with “yes,” “no,” or “cannot determine” to those questions. Study quality was rated as “good” when assessors answered “yes” to 10 or more questions; “adequate” in case of seven, eight, or nine “yes” answers; or “poor” in case of less than seven “yes” answers. Quality assessment of qualitative studies was based on the checklist from the Critical Appraisal Skills Programme (Critical Appraisal Skills Programme, 2018). The CASP Qualitative Checklist requires assessors to answer “yes,” “no,” or “cannot tell” to 10 questions relating to whether research aims are clearly stated, to the appropriateness of methodology, study design, recruitment strategy and data collection method, to the consideration of the relationship between researchers and participants, ethical considerations, rigor in data analysis, clarity of presentations of study findings, and overall value of the research. As proposed by Lehane et al. (2017a), studies received a “good” quality rating when assessors answered “yes” to eight or more questions, an “adequate” rating for six or seven “yes” answers, and a “poor” rating for less than six “yes” answers. Mixed methods studies were rated according to both assessment tools, and assessors discussed the overall quality rating until consensus was reached. As the aim of this review was to integrate evidence across research designs, disciplines, and health impairments, the authors did not limit reporting of results to studies with better quality assessment.

## Results

**Figure 1:**
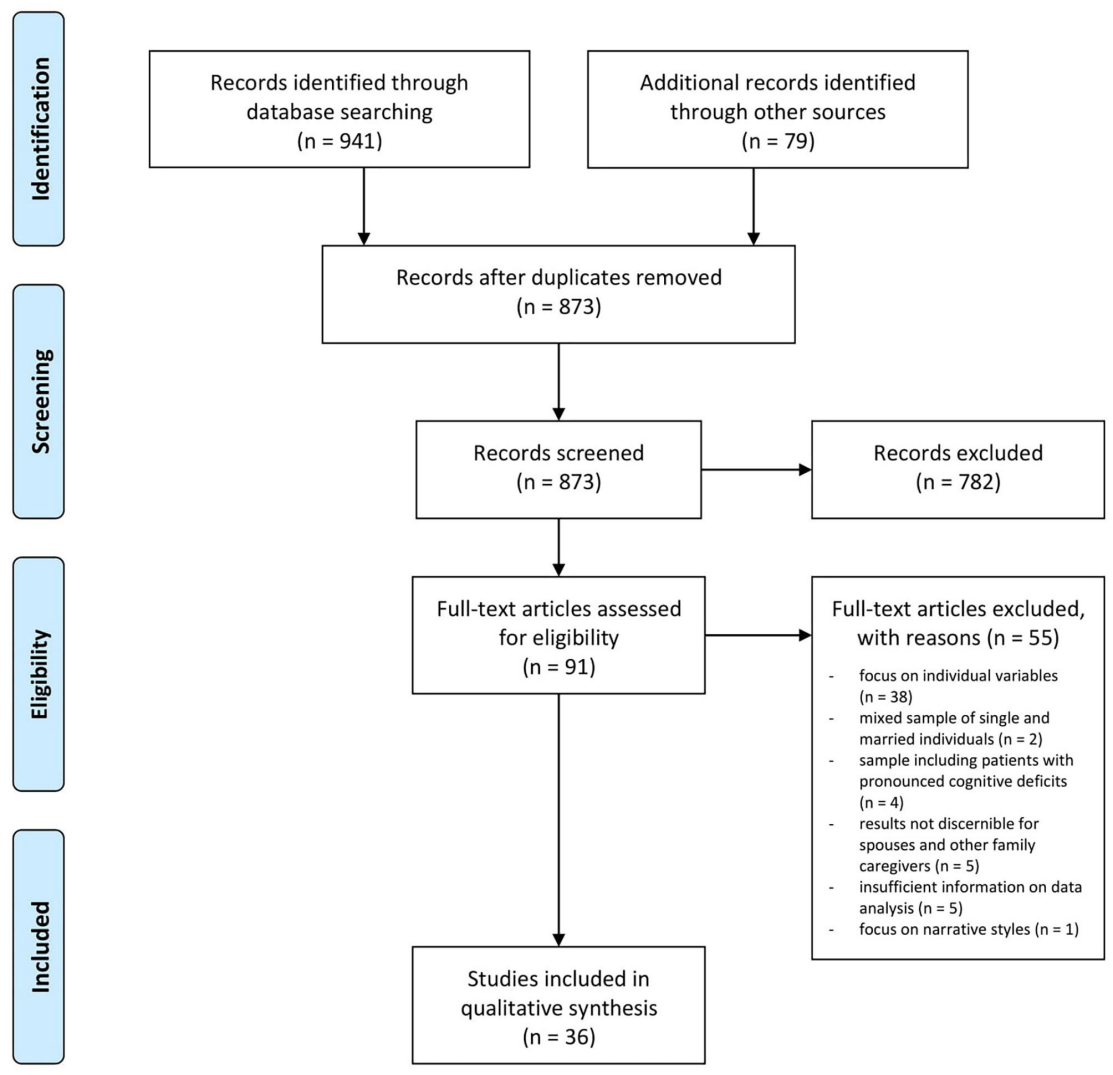
PRISMA flow diagram: study retrieval and selection.

The included articles contained a high proportion of qualitative studies (64%) that focused on the processual nature of coping with one partner's impairment over time. As such, these studies reported findings relevant to both review aims of identifying (1) dyadic challenges in couples confronted with physical or sensory impairment and (2) dyadic coping with these impairments. To avoid redundancy, the authors decided to merge the search results of both literature searches and conduct a unified analysis for both research questions. A flowchart illustrating the article selection process for the unified analysis is provided in Figure 1. The initial database searches yielded a total of 941 articles for screening. After removal of duplicates and addition of results from the hand searches, the titles and abstracts of 873 articles were screened. Of those, 91 were retained for eligibility screening of full texts. Fifty-five full texts were excluded (see detailed reasons in Figure 1). The final number of included articles was 36.

### Study Characteristics and Health Impairments

Detailed characteristics of studies included in this review can be found in Table 1. Two included articles were reviews. One review summarized evidence on couples coping with stroke in the community (Ramazanu et al., 2020), and the other review focused on the consequences of sensory loss for couples' well-being (Lehane et al., 2017a). Overlap between included studies with the current review was evident for five studies (see Table 1). The two reviews, within their respective health impairment focus, had a broader scope than the present review and included also studies with couples reporting on individual outcomes such as individual psychological well-being. In the present results section, we will report only on results related to dyadic processes or outcomes retained in the two reviews. Of the empirical studies included in this review, 23 applied a qualitative design, 2 used mixed methods, and 10 applied quantitative methodology. Cross-sectional designs (*n* = 29) were more frequent than longitudinal designs (*n* = 5). Samples sizes ranged from *N* = 2 to *N* = 320 individuals. In seven manuscripts, raters identified quality concerns that led to an overall quality assessment of “poor” (see Table 1). Most “poor” quality assessments were due to the use of cross-sectional data in quantitative studies and concerned studies from the 1990s. The most frequent quality concern in qualitative studies was the lack of a critical discussion of the role of the researchers during all stages of the research. In the following, all included articles will be considered for the synthetization of the results.

**Table 1:** Characteristics and findings of empirical studies included for review.

**References**	**Health impairment**	**Participants**	**Design and data collection**	**Measures/interview topics**	**Findings**	**Quality rating (study type)**
**Sensory impairments**						
Burton et al. (2015)	Vision loss due to AMD	1 couple, both partners with AMD and comorbidities Ages: 82 and 77 years RD: 60 years	Longitudinal case study with 3 time points Semistructured joint interviews	Diagnosis and couple life since, daily activities, thoughts about the future, relationship	The couple had to adjust everyday activities and manage mutual loss of independence. There were no references to enjoyment in everyday activities. Couple demonstrated a sense of “we” and experienced resilience and unity due to sharing a diagnosis.	Adequate (qualitative)
Lehane et al. (2018)	Dual-sensory loss	45 spouses *M*_age_: 69.21 years *M*_RD_: 71.7 years *M*_TSD_ patients: 20.3 years	Cross-sectional Questionnaire	Couples' Illness Communication Scale, Couples' Satisfaction Index, Medical Outcomes Study Social Support Survey	Significant association between sensory-loss-related communication, RS, perceived support, and psychological well-being. Perceived support mediated the association between communication and well-being.	Adequate (quantitative)
Glade (2018)	Hearing loss	6 couples Age range of patients: 60–79 years	Cross-sectional Semistructured individual interviews	Communication with spouse and in social situations prior to and after cochlear implants (CI), experience with auditory rehabilitation	Prior to use of cochlear implants, communication caused frustration and tension within the couples and impeded satisfying social interactions. Social interactions improved following cochlear implant, but adjustment was an extended process.	Good (qualitative)
Scarinci et al. (2008)^a^	Hearing loss	10 spouses *M*_age_: 70.2 years *M*_RD_: 44.6 years	Cross-sectional Semistructured individual interviews	N/A	The partner's hearing impairment meant overall reduction in communication, frequent communication breakdown, increased relationship tension, reduced time spent together, and less opportunities for experiencing togetherness.	Adequate (qualitative)
Yorgason et al. (2007)^a^	Hearing loss	8 couples *M*_age_ of patients: 68 years	Cross-sectional Semistructured joint interviews	Relational experiences surrounding hearing loss, meaning of hearing loss, what could help the couple thrive in their relationship despite impairment	Hearing-related stressors included negative emotions and conflict related to impaired hearing, reduced communication opportunities and embarrassment in group settings, and loss of shared activities. Couples experienced resilience through individual and dyadic meaning-making and attunement to needs for interdependence and autonomy.	Adequate (qualitative)
**Physical impairments**						
Zhaoyang et al. (2018)	Arthritis	T1: 142 couples; T2: 132 couples *M*_age_ of patients at T1: 65.78 years *M*_RD_ at T1: 34.71 years *M*_TSD_ at T1: 16.42 years	Longitudinal Questionnaire	Items on disclosure and holding back adopted from Porter et al. (2008), Dyadic Adjustment Scale	Holding back at T1 was associated with decreases in own RS in patients and partners. Increases in disclosure were associated with increases in own RS. No partner effects from holding back or disclosure on partner RS.	Good (quantitative)
Schembri Lia and Abela (2019)	Locomotor disability	3 couples RD range: 23–47 years	Cross-sectional Semistructured individual and joint interviews	N/A	Couples showed sensitivity and attunement to each other's feelings and needs and had a clear vision of remaining together.	Adequate (qualitative)
					Major struggles included altered sexual intimacy and unease with imbalance in support provision.	
Blackmore et al. (2011)	MS	81 patients *M*_age_: 46.9 years *M*_TSD_: 10.3 years	RCT; pre-/post-design Questionnaire	Sexual Disabilities section of Guy's Neurological Disability Scale, UCLA Social Support Scale, Sexual Satisfaction Inventory	Increases in perceived positive partner support and decreases in negative partner support were associated with improvements in sexual satisfaction.	Adequate (quantitative)
Boland et al. (2012)	MS	7 couples Median age of patients: 53 years Median RD: 29 years Median TSD: 10 years	Cross-sectional Semistructured individual interviews	Description of own coping approach, changes/adjustment in coping over time	Coping with MS had ups and downs and couples constantly needed to bring their coping efforts in sync. Coping occurred over a long time and changed depending on disease stage. Maintaining faith that the relationship was worthwhile independent of the changes helped couples cope.	Good (qualitative)
Ghafari et al. (2014)	MS	25 patients *M*_age_: 38 years *M*_TSD_: 9 years	Cross-sectional Semistructured individual interviews, field notes	Relationship with partner, partner's support to adapt to disease	Patients expressed a higher need for emotional support than instrumental support while they perceived spouses to provide mainly instrumental support. They strove for functional independence to maintain a balance between partners. Mutual understanding helped create and maintain a satisfying relationship despite inevitable changes.	Good (qualitative)
Samios et al. (2015)	MS	T1: 160 couples; T2: 98 couples *M*_age_ of patients at T1: 49.65 years *M*_TSD_: 10.43 years	Longitudinal Questionnaire	Dyadic Adjustment Scale	RS decreased from T1 to T2. Patient and partner RS were significantly related at T1 and T2. Significant partner effects from RS T1 to RS T2.	Good (quantitative)
Starks et al. (2010)	MS	8 couples Age range of patients: 40–69 years RD range: 1.2–47 years TSD range: 1–21 years	Cross-sectional Questionnaire, semistructured individual and joint interviews	Perceived Social Support Scale, Dyadic Adjustment Scale	Four couples were “in-sync,” i.e., had compatible world views and communication styles, worked together to solve challenges moved forward together. Four couples were “out-of-sync,” i.e., had contrasting coping styles, focused on different priorities and adjusted at different paces, but were committed to the relationship. Patients from in-sync couples had longer time since diagnosis, mostly gradual onset of MS and retained high levels of independence.	Adequate (mixed methods)
Wawrziczny et al. (2019)	MS	6 couples *M*_age_ of patients: 39.17 years *M*_RD_: 17.17 years *M*_TSD_: 8 years	Cross-sectional Semistructured joint interviews	Experience of disease, relationship history, changes in relationship and adjustments in daily life since disease onset, social support	Disease progression made couples' lives increasingly revolve around MS. Different challenges for patients and spouses and their inability to mutually share and validate each other's experience led to withdrawal and alienation.	Adequate (qualitative)
Carter and Carter (1994)	PD	Group A: 20 PD patients, 20 ill spouses; group B: 26 PD patients, 26 well spouses *M*_age_ of patients: 65.7 years *M*_RD_: 39.3 years	Cross-sectional Questionnaire, sentence completion task	Projective Sentence Completion, Dyadic Adjustment Scale	No group difference on marital adjustment. Cohesion in PD couples higher than norms, consensus lower. Effects of illness on marriage mostly positive, good marriage considered essential in PD.	Poor (quantitative)
Martin (2016)	PD	21 patients, 23 spouses *M*_age_: 67 years *M*_RD_: 38 years *M*_TSD_: 6 years	Cross-sectional Semistructured individual interviews	Impact of PD on self, partner and relationship	The main relational stressors implied by PD included financial strain, shifts in relational roles, changed sexual intimacy and overall closeness between partners, less leisure and social activities, and resulting uncertainty about whether the relationship would continue.	Adequate (qualitative)
Smith and Shaw (2017)	PD	4 couples, 1 widowed spouse Age range: 67–85 years TSD range: 2–21 years	Cross-sectional Semistructured individual interviews	Reactions to diagnosis, life changes due to PD	PD put strain on relationships, especially due to changes in responsibilities for tasks, but made participants realize how much they valued their relationships. Couples adjusted best when they assimilated PD and retained agency despite difficult changes.	Poor (qualitative)
Wootton et al. (2019)	PD	9 couples RD range: 4–45 years	Cross-sectional Semistructured individual interviews	Relationship and health history, experiences and relational impact of facial masking, coping with the impacts	Patients' muted and slowed facial expressions led to partners' difficulties understanding intentions and feeling. They were often misinterpreted as disinterest in the relationship and led to emotional distance and disconnection. To counteract, couples used more touch and gesture and verbal communication to clarify misunderstandings.	Adequate (qualitative)
Chan (2000)	SCI	66 patients, 40 spouses *M*_age_ of patients: 45.18 years *M*_TSI_: 13.27 years	Cross-sectional Semistructured individual interviews	Impact of SCI on family and marital relationships, sources of stress, life satisfaction, caregiving burden	Relationship stressors included financial strain, role changes and worries about the future, increased conflict, changes in feelings (love to sense of care, sympathy), difficulties communicating about needs, and reduced social circle. Maintenance of marriage was “no stress-free process”; required mutual understanding and support, patience and acceptance of disability and its consequences.	Adequate (qualitative)
Dickson et al. (2010)	SCI	11 spouses *M*_age_: 51.4 years Mean time as caregiver: 6.5 years	Cross-sectional Semistructured individual interviews	Experience of becoming a spousal SCI caregiver, life changes due to caregiver role	Participants reported a sense of loss of their partner and pre-injury life. They experienced drastic role changes from spouse and lover to parental caregiver figure, especially linked to loss of physical intimacy. Appreciation for each other increased and contributed to improved relationships in some couples.	Good (qualitative)
Engblom-Deglmann and Hamilton (2020)	SCI	11 couples	Cross-sectional Semistructured individual and joint interviews	Most significant stressors in marriage, initial cognitive processes following injury, coping with losses related to SCI, positive impact of SCI on relationship	Central challenges for couples were altered sexual function, negotiation of care needs and social disconnection following SCI. Adaptability in couples ranged from connection/flexibility to constriction/stagnation.	Good (qualitative)
Freeman et al. (2017)	SCI	5 couples *M*_RD_: 16 years	Cross-sectional Semistructured joint interviews	Couple's experience of inpatient rehabilitation (IR) and its influence on relationship, strategies to maintain relationship, intimate and sexual expression during IR	Couples emphasized being a unit and expressed disappointment about healthcare staff who did not acknowledge them as a dyad. Physical and emotional fatigue and loss of spontaneity meant that sexual intimacy was not a priority during inpatient rehabilitation.	Adequate (qualitative)
Jeyathevan et al. (2019)	SCI	19 patients, 15 family caregivers (9 spouses, 6 parents) Age range of patients: 22–65 years	Cross-sectional Semistructured individual interviews	Changes in relationship post-injury, adjustment of family to SCI, impact of SCI on family roles, handling of sex and intimacy post-injury, perceived affectedness of caregiver	In some cases, post-injury relationships deteriorated due to asymmetrical dependencies, protective behaviors of caregivers and loss of sexual and emotional intimacy. Relationships were maintained or rebuilt when partners were interdependent, creatively shifted commonalities and routines, i.e., when they established a “new normal.”	Good (qualitative)
Kreuter et al. (1994)	SCI	49 spouses Median age: 34 years Median RD: 6 years Median TSI: 5.5 years	Cross-sectional Questionnaire	Self-designed Sexual Interest, Activity and Satisfaction Scale, Sexual Behavior Scale, Emotional Quality of the Relationship Scale	Majority of spouses satisfied with relationship and current sex life, although almost half of the sample reported decline in sexual activity. One third reported problems discussing sex with their partner.	Poor (quantitative)
Yim et al. (1998)	SCI	30 SCI couples; 30 able-bodied couples *M*_age_ of patients: 39.80 years *M*_RD_: 12.90 years	Cross-sectional, group comparison Questionnaire	Short Marital Instability Scale, culturally adjusted Dyadic Adjustment Scale, Marital Agendas Protocol	No significant group difference between marital adjustment and RS. Cohesion and marital stability higher in SCI couples. Sex as the most serious problem in SCI couples.	Poor (quantitative)
Anderson et al. (2017)^b^	Stroke	18 couples *M*_age_ of patients: 62.6 years *M*_RD_: 34.4 years	Cross-sectional Semistructured individual or joint interviews	Couples' history, current roles, current relationship, strategies to make marriage work, immediate post-stroke experience	Satisfied couples reported having adequate resources to reconstruct role identities, good discussions and focusing on love while dissatisfied couples experienced role overload and disengagement and reported mutual insensitivity to each other's feelings and lack of listening.	Good (qualitative)
Croteau et al. (2020)	Stroke	9 couples *M*_age_ of patients: 69 years RD range: 27–63 years TSD range: 1.1–7.6 years	Cross-sectional Semistructured individual interviews	Modes and frequency of communication before and after stroke, content of conversations	Most participants reported a decrease in the frequency, duration, and variability of conversations. Communication became associated with negative emotions due to difficulties. Spouses took on a speaker role, patients adopted a listener role, with difficulty establishing equilibrium in conversation.	Good (qualitative)
Korpelainen et al. (1999)	Stroke	192 patients, 94 spouses *M*_age_ of patients: 59.1 years Median TSD: 23 months	Cross-sectional Questionnaire	Self-designed items for sexual function and explanatory factors	Decreased libido in more than half of patients and spouses. Marked increase in sexual dissatisfaction post-stroke.	Poor (quantitative)
McCarthy and Bauer (2015)^b^	Stroke	31 couples *M*_age_ of patients: 61.81 years *M*_RD_: 26.09 years *M*_TSD_: 9.23 years	Cross-sectional Unstructured individual interviews	Ways in which stroke has disrupted own life, spouse's life and couple relationship	Stroke marked a disruption and pausing of normal life course. Relationship challenges included compromised physical intimacy, shifts in marital roles, social isolation, and uncertainty about the future due to perceived unpredictability. Couples with shorter relationship duration handled role changes better. Couples drew on existing relationship strengths to cope.	Adequate (qualitative)
Quinn et al. (2014)^b^	Stroke	8 couples Age range of patients: 36–61 years *M*_RD_: 26 years *M*_TSD_: 4.5 years	Cross-sectional Semistructured joint interviews	Pre-stroke relationship, immediate experience following stroke, life and relationship changes post-stroke	Couples reported a transition to roles as carer and cared for, for some adopting characteristics of a parent–child relationship. Both partners were reluctant to fully accept the changed roles.	Good (qualitative)
Robinson-Smith et al. (2016)	Stroke	EG: 5 couples; CG: 5 couples *M*_age_ of patients: 65.2 years	Pilot intervention study, pre-/post-design Questionnaire, field notes from home visits	Dyadic Coping Inventory; field notes on couples' thoughts and feelings regarding post-stroke relationships	Dyadic coping by oneself increased in stroke patients following intervention. Positive dyadic coping increased in EG spouses. Patients reported changes in roles and reciprocity between partners. Attempts at maintaining intimacy included talking and reminiscing more	Poor (mixed methods)
Schmitz and Finkelstein (2010)	Stroke	15 patients, 14 spouses Median age of patients: 65 years Median TSD: 45 months	Cross-sectional Semistructured individual interviews	Experience of having stroke, sexuality after stroke, discussion of sexuality with rehabilitation professionals	Decreased sexual desire or activity post-stroke were linked to physical and emotional challenges, disrupted roles within relationship and discomfort discussing sex with the partner.	Good (qualitative)
Bodley-Scott and Riley (2015)	TBI	5 spouses Age range of patients: 29–42 years RD range: 6–22 years TSI range of patients: 0.75–7 years	Cross-sectional 1 narrative and 1 evaluative semistructured interview per participant	Account of partner's injury and subsequent changes, evaluation of relationship changes	Spouses experienced direct negative emotional impact of partner's TBI associated with sense of loss for their “old” partner. Shift from lovers to carer and care recipient led some to consider ending the relationship. Love was replaced by a sense of care. Loss of sexual intimacy and shared enjoyment contributed to emotional distance.	Good (qualitative)
Kreutzer et al. (2016)	TBI	42 couples *M*_age_ of patients: 49.8 years *M*_TSI_: 2.2 years	Cross-sectional Questionnaire	Marital Status Inventory, Revised Dyadic Adjustment Scale	24% of patients and 29% of spouses considered their marriage as unstable. Half of the sample reported clinically significant levels of marital dissatisfaction.	Poor (quantitative)
O'Keeffe et al. (2020)	TBI	5 patients, 6 spouses RD range: 9–32 years TSI range: 4–8 years	Cross-sectional Semistructured individual interviews	Perceptions of changes, challenges, and positive aspects of relationship post-injury	Both partners experienced a sense of loss regarding pre-injury relationship caused by role changes, altered communication, increased conflict, reduced sexual intimacy and emotional connectedness. Couples negotiated a new equilibrium based on respect, loyalty, understanding, and hope.	Good (qualitative)

*AMD, age-related macular degeneration; CG, control group; EG, experimental group; N/A, information not available; M, mean; MS, multiple sclerosis; RD, relationship duration; RS, relationship satisfaction; SCI, spinal cord injury; TBI, traumatic/acquired brain injury; TSD, time since diagnosis; TSI, time since injury*.

a *The study was also included in the review by Lehane et al. (2017a)*.

b *The study was also included in the review by Ramazanu et al. (2020)*.

Most studies (*n* = 34) focused on a single health condition. The health conditions primarily related to physical disability were stroke (*n* = 8), spinal cord injury (SCI; *n* = 7), multiple sclerosis (MS; *n* = 6), Parkinson's disease (PD; *n* = 4), traumatic/acquired brain injury (TBI; *n* = 3), and arthritis (*n* = 1). Studies on sensory disability investigated hearing loss (*n* = 3), vision loss due to age-related macular degeneration (*n* = 1), and dual-sensory loss (*n* = 1). Only two studies used combined samples: different locomotor disabilities in one case and mixed sensory loss (i.e., vision loss, hearing loss, and dual-sensory loss) in the other case. Further details on study characteristics, e.g., developmental-contextual factors such as mean relationship duration or time since diagnosis, can be found in Table 1.

### Dyadic Challenges Related to the Health Impairment

**Figure 2:**
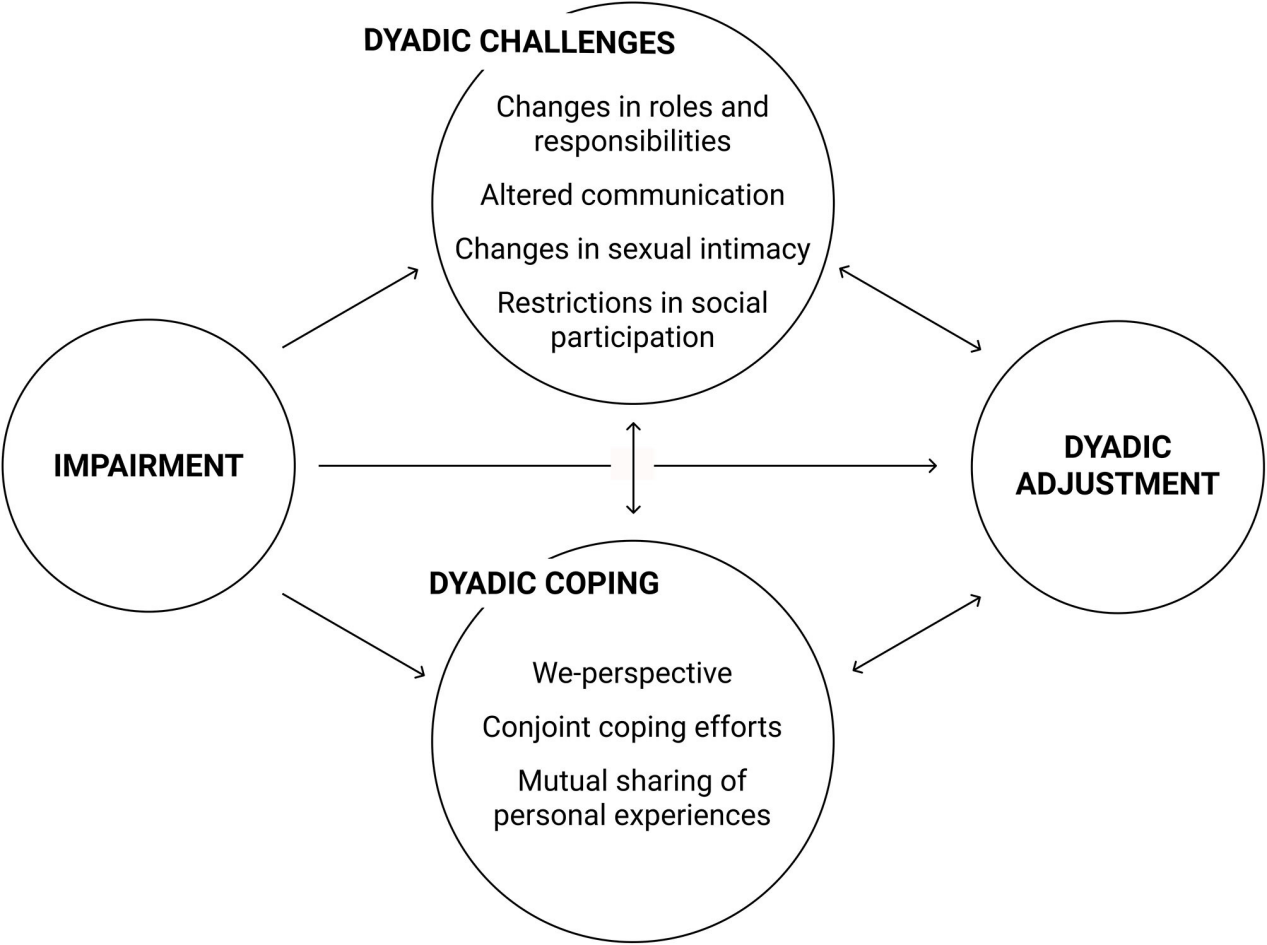
Interrelations of dyadic challenges, dyadic coping, and dyadic adjustment in couples facing chronically disabling physical or sensory impairments.

Both reviews and most studies supported the notion that disability was an interpersonal experience. The relationship impact of physical or sensory impairments identified across all the reviewed studies was substantial. This was evident, for instance, in one of the five themes identified by Ramazanu et al. (2020) review about couples coping with stroke: Marital relationships were found to be “at a point of change” (p. 479) in the majority of couples. Similarly, Smith and Shaw (2017) paraphrased their participants' experience of PD as “learning to live in a new way” (p. 16)—not only for the patient, but for the couple as a unit. Changes across different areas of the relationship challenged couples to renegotiate established patterns of interaction. Most consistently reported challenges across health impairments were changes in roles and responsibilities, altered communication, changes in sexual intimacy, and restrictions in social participation. Figure 2 illustrates these dyadic challenges and how they interact with dyadic coping to contribute to dyadic adjustment according to the findings of the current review.

#### Changes in Roles and Responsibilities

Changes in roles and responsibilities were reported across all health impairments. Some patients were forced into early retirement or to significantly reduce work hours due to their ill health. When the patient had been the primary provider within the couple, this resulted in substantial shifts in responsibility for generating family income (Chan, 2000; Robinson-Smith et al., 2016; Engblom-Deglmann and Hamilton, 2020). As was shown in couples coping with stroke, shifts in marital roles were especially challenging for couples who had been together for a long time, i.e., who had more firmly established roles. Also, the patient's sudden inability to work for pay was more of an issue for working-age than for elder couples (McCarthy and Bauer, 2015). Some spouses reported experiencing role overload due to the sudden surge in responsibilities inside and outside the couple's home. Role overload was reduced when the couple had the financial means to pay for professional assistance (Anderson et al., 2017).

An imbalance between the two partners was often described. Such imbalance may be inherent in the notion of role overload in that one partner adopts different roles and associated tasks while the other partner, usually the patient, is forced to renounce past roles. For instance, Jeyathevan et al. (2019) used the theme “asymmetrical dependency” to describe the relationship of individuals with SCI and their family caregivers. The couples in the study of Dickson et al. (2010) referred to the “one-sidedness” of many couple interactions post-SCI. Stroke survivors described changes in reciprocity between them and their spouses (Robinson-Smith et al., 2016). Inequities in giving and taking were also evident as a stressor across the studies included in a review on couples coping with sensory loss (Lehane et al., 2017a). In patients, the perceived imbalance between partners was reported to cause feelings of guilt (Schembri Lia and Abela, 2019), fears of being perceived as a burden (Robinson-Smith et al., 2016; Anderson et al., 2017), and a perceived threat to autonomy (McCarthy and Bauer, 2015). A sense of loss of control over their own life also threatened the autonomy of partners (Dickson et al., 2010) and was more pronounced in couples with shorter relationship duration (McCarthy and Bauer, 2015).

The most stressful change of roles for most couples was the perception of transitioning from romantic partners to caregiver and care recipient. The majority of partners in Engblom-Deglmann and Hamilton (2020) study talked about the “transition from lover 1 day to caregiver the next day” being very difficult. A seeming incompatibility of the romantic partner and caregiver roles was evident across health impairments, e.g., in couples coping with stroke (Quinn et al., 2014; McCarthy and Bauer, 2015; Ramazanu et al., 2020), TBI (Bodley-Scott and Riley, 2015), and PD (Martin, 2016). Some couples described that their relationships had, to a varying degree, adopted the dynamics of a parent–child relationship (Dickson et al., 2010; Quinn et al., 2014). This was in part due to substantial changes in the couples' sexual intimacy which will be discussed below.

The vast majority of studies concluded that changes in roles and responsibilities were perceived as stressful by couples coping with physical or sensory impairments. For instance, the perception that partners and patients experienced very different struggles in coping with MS depending on their roles led some couples to withdraw. Coupled with an inability to openly discuss differing experiences, this ultimately increased emotional distance (Wawrziczny et al., 2019). Similarly, many couples reported that the changes in roles and responsibilities meant losing their pre-disability relationship. This sense of loss was associated with negative emotions (e.g., O'Keeffe et al., 2020). It should, however, be noted that one study on the caregiving experience in SCI explicitly mentioned that some individuals perceived changes in roles and responsibilities as beneficial because they were perceived to rebalance asymmetries that had existed between the partners prior to the injury (Chan, 2000).

#### Altered Communication

Several health conditions or subsequent impairments reviewed here directly impact on communicative abilities. For instance, hearing loss strongly affects speech comprehension as an important part of verbal communication. Consistently, all three qualitative studies focusing on couples coping with hearing loss reported difficulties in couples' communication due to the hearing impairment (Yorgason et al., 2007; Scarinci et al., 2008; Glade, 2018). Scarinci et al. (2008) summarized these difficulties in the theme “You can't carry on a normal conversation” (p. 144) and mentioned the following effects of hearing impairment on couples' communication: the perception of verbal communication as tiring and unenjoyable; reduced amount of prolonged conversations between partners; reduced spontaneous verbal interactions such as short, trivial remarks; and the inability to share secrets. The review of Lehane et al. (2017a) on the consequences of sensory loss for couples also concluded that communication difficulties and misunderstandings were frequent and might be related to both individual and dyadic adjustment, e.g., feelings of frustration or withdrawal from couple interactions.

While in sensory loss, the associations between impairment and communication are straightforward, characteristic presentations of physically disabling health conditions may not appear to directly relate to communicative abilities. However, there are symptoms of these conditions that do impact couples' communication. For example, in PD patients, hypomimia or facial masking describes a decrease in voluntary control and spontaneous movement of the muscles in the face. As Wootton et al. (2019) showed, muted and slowed facial expressions by PD patients made it hard for spouses to read facial expression. Muted and slowed facial expressions reduced the availability of non-verbal cues that would be used to make inferences about emotional content or intentions of the patient. This, in turn, was reported to lead to misunderstandings that were frustrating for both partners and contributed to an increase in emotional disconnection. Croteau et al. (2020) focused on a subset of stroke couples where patients presented with chronic stroke-related aphasia, i.e., impairments in language comprehension and/or production. Couples reported that the frequency and duration of their conversations had decreased due to aphasia, conversational topics were narrowed down, conversations became more superficial, and patients participated less in conversation than pre-stroke. Although not all couples provided a negative account of their post-stroke communication, communication changes were evident for most couples.

#### Changes in Sexual Intimacy

Changes in sexual intimacy due to impaired health are reported in many studies. Generally compromised physical intimacy was reported in couples facing SCI (Engblom-Deglmann and Hamilton, 2020), stroke (McCarthy and Bauer, 2015), and TBI (Bodley-Scott and Riley, 2015). More specifically, almost all spouses of individuals with SCI reported sexual functioning of the patient had been altered post-injury. One third of the spouses wished for more frequent sexual activity. However, almost half of the spouses considered their sex life post-injury to be as good as or better than pre-injury (Kreuter et al., 1994). More than half of participants who had divorced a person with SCI post-injury named decreased sexual ability post-injury as the main cause for their divorce (Chan, 2000). Similarly, stroke patients and spouses of stroke patients both reported marked declines in their libido and sexual activity following the stroke. Sexual dissatisfaction was reported by 49% of patients and 31% of spouses. Functional disability, unwillingness to participate in sexual activity, and an unease to discuss sexuality with the partner were significant predictors of sexual dissatisfaction in these stroke couples (Korpelainen et al., 1999). Interviews with stroke patients and their romantic partners supported the finding that most participants experienced decreases in sexual desire or activity. They linked this decrease to physical and emotional challenges, e.g., erectile dysfunction and fear of sexual activity causing another stroke. However, participants stressed their continued need for touch and emotional connection (Schmitz and Finkelstein, 2010).

Besides functional disability, the suggested pathways through which physical and sensory disability affect sexual intimacy in couples mostly referred to complex changes in the couple relationship. Three of the 10 interviewed spouses of individuals with hearing impairment reported a reduction in intimate talk and increased tension, which ultimately affected their sexual relationships (Scarinci et al., 2008). The role changes perceived by many couples across health impairments were also important contributors to reduced sexual intimacy. Some spouses reported experiencing role conflicts between being a caregiver and being a romantic, sexual partner (Jeyathevan et al., 2019; O'Keeffe et al., 2020). The loss of a sexual relationship caused some spouses in SCI couples to feel like their role had changed to a parental role, underlining the interrelations between role changes and sexual intimacy changes (Dickson et al., 2010). In general, global changes in the couple relationship and dyadic interactions were more important for sexual satisfaction than functional ability or individual well-being (Blackmore et al., 2011). For example, support transactions between partners also seem to affect sexual intimacy. MS patients who had received telephone-administered psychotherapy for depression reported improvements in sexual satisfaction when positive partner support had increased from baseline to post-treatment and when negative partner support had decreased. This remained true when controlling for sexual dysfunction and depression severity (Dickson et al., 2010).

Another contributor to struggles in maintaining sexual intimacy was insecurities in partners around mutual attractiveness. Females with locomotor disability reported struggling to feel feminine and sexually attractive for their partners (Schembri Lia and Abela, 2019). Conversely, their spouses reported difficulty feeling sexual attraction when they saw their partner experiencing physical pain and perceived them as fragile. Similar accounts were reported in couples coping with PD. Insecurities of the patients whether they remained attractive as partners caused some couples to feel less secure about the stability of their relationship and thus less close to their spouses, emotionally and sexually (Martin, 2016).

Lastly, structural barriers to a fulfilling expression of intimacy including sexual intimacy were reported by Freeman et al. (2017). Focusing on couples' experiences of relationship maintenance during acute SCI rehabilitation, they found that the inpatient environment limited couples' privacy and, thus, their opportunities to express intimacy. While couples perceived themselves as a unit going through rehabilitation, healthcare professionals were reported to engage in behavioral patterns that undermined the couples' sense of unity.

#### Restrictions in Social Participation

While the challenges posed by the health impairment had an impact on the couple relationship as such, it also affected the couple's opportunities to jointly participate in social life outside the home. A general feeling of being “isolated from the broader world” was reported for younger couples coping with stroke (McCarthy and Bauer, 2015). SCI was related to reduced social esteem and thus reduced social circle in couples from Hong Kong (Chan, 2000). Dickson et al. (2010) reported that some spouses of individuals with SCI felt they had become invisible to other people following their spouse's injury. Similarly, some SCI couples reported increased social disconnection post-injury due to accessibility issues and because they experienced friends to feel uncomfortable interacting with the couple (Engblom-Deglmann and Hamilton, 2020). Accessibility issues were also reported to reduce opportunities to socialize with friends in PD couples (Martin, 2016). Restrictions in social participation were also an important issue in couples coping with sensory loss. Communication problems seemed to induce embarrassment in social situations leading the couples to socialize less (Yorgason et al., 2007; Scarinci et al., 2008; Lehane et al., 2017a). In hearing loss, cochlear implants were reported to improve social interactions that had been difficult pre-implant (Glade, 2018). One strategy to counteract a lack of social participation was for partners of individuals with SCI to establish social lives separate from their partners. This, however, meant a loss of shared activities for the couple (Engblom-Deglmann and Hamilton, 2020). Spouses of TBI patients reported that a reduction in opportunities for shared enjoyment contributed to an increasing distance between them and their partners (Bodley-Scott and Riley, 2015). Loss of shared activities and spending less time together was also reported in couples coping with hearing loss (Yorgason et al., 2007; Scarinci et al., 2008) and PD (Martin, 2016) and contributed to a decrease in closeness of the partners.

### Dyadic Coping

Dyadic coping reported in the included studies was found to help buffer the stress couples experience due to chronically impaired health (see Figure 2). In the following, the most helpful dyadic coping strategies reported by couples are presented.

#### Mutual Sharing of Personal Experiences

Holding back from disclosing personal experiences seemed to be a prevalent phenomenon in couples coping with physical and sensory disability of one partner. Studies suggested that avoidance of certain topics, cautious communication and holding back, feeling uncomfortable sharing one's emotions, and protective buffering were common in couples coping with SCI (Chan, 2000; Jeyathevan et al., 2019), stroke (Croteau et al., 2020), MS (Wawrziczny et al., 2019), and TBI (O'Keeffe et al., 2020). Holding back from sharing personal experiences seemed to be relationship-compromising. In a longitudinal dyadic study on knee osteoarthritis (OA), patients and their spouses reported the extent to which they disclosed or held back from discussing their concerns with their partner. Holding back concerns regarding symptoms and treatment, activity limitations due to OA, disease progression, own negative feelings, relationship with the spouse and others, and financial strain was associated with decreases in one's relationship satisfaction over a 1-year period for both patients and spouses (Zhaoyang et al., 2018).

While holding back from sharing personal experiences was relationship-compromising, mutually sharing personal experiences appeared to be relationship-enhancing. In the OA sample, increased disclosure of concerns was associated with increases in relationship satisfaction over the course of 1 year (Zhaoyang et al., 2018). Higher scores of mutual sensory loss-related communication were also positively associated with relationship satisfaction in spouses of individuals with dual-sensory loss. Perceived reciprocity in spouses' willingness to discuss sensory loss together was also associated with perceived support, suggesting partners' willingness to share their experiences of sensory loss contributed to the spouse's feeling of being cared for in the relationship (Lehane et al., 2018). Similarly, MS patients considered the ability to talk with their spouses about personal difficulties and needs essential to establishing and maintaining a comforting relationship (Ghafari et al., 2014). Couples who were found to be satisfied with the reconstruction of their relationships after one partner's stroke reported how they had continued or learned to talk together about their difficulties and needs following stroke. In contrast, dissatisfied couples seemed to remain stuck in patterns of mutual holding back and withdrawal from communication (Anderson et al., 2017).

#### We-Perspective and Conjoint Coping Efforts

Couples who adopted a “we-perspective” with regard to coping with the consequences of the health impairment seemed to adjust well. The couples' sense of togetherness helped them cope with stressors associated with the disease. For example, Boland et al. (2012) noted that the MS couples in their study shared a perception that “they would cope better together than if they were separated” (p. 1,371). Both partners' perception that they were “in it together” was also named as an important factor in maintaining or re-establishing satisfying relationships in couples coping with vision loss (Burton et al., 2015) and SCI (Freeman et al., 2017). For instance, individuals with SCI and their family caregivers both emphasized the need to mutually rely on each other and their joint responsibility to rebuild the relationship post-injury. They considered both relationship partners to be interdependent, reflecting a strong we-perspective (Jeyathevan et al., 2019). Conversely, in couples coping with MS, a main finding was that each spouse withdrew and fought the disease individually. These couples lacked a we-perspective and did not engage in conjoint coping efforts. The participating couples were described as “alienated,” indicating that their individual approaches to coping took a toll on the relationship (Wawrziczny et al., 2019). Similarly, mutual withdrawal or disengagement from coping contributed to feelings of disconnection and a loss of we-perspective in TBI couples (O'Keeffe et al., 2020), PD couples (Wootton et al., 2019), and partners of individuals with SCI (Dickson et al., 2010). Accordingly, MS couples who were considered “in-sync” by Starks et al. (2010, p. 198) were portrayed to frequently work as a team in relation to problem-solving while “out-of-sync” couples only rarely worked as a team. Conjoint coping efforts were also important for re-establishing relationship satisfaction following stroke. Couples who reported mutual awareness of each other's feelings and resolved conflict by discussing problems together adjusted best. Such efforts to understand each other's experience and act in the best interest of the couple reflected a we-perspective in partners. In contrast, couples from the same study who had divorced or remained married despite considerable dissatisfaction described how mutual unwillingness to learn about each other's experiences contributed to increasing escalation of conflicts (Anderson et al., 2017).

#### Factors Favoring and Hindering Mutual Sharing, We-Perspective, and Conjoint Coping

Some studies indicated characteristics and processes that favored the positive forms of dyadic coping summarized above, i.e., mutual sharing, we-perspective, and conjoint coping. Firstly, participants from several studies emphasized the relevance of pre-impairment relationship quality. Couples coping with locomotor disability of the wife all pointed out that a strong relationship basis prior to the development of the wife's impairments was paramount to adjusting well as a couple (Schembri Lia and Abela, 2019). Similarly, PD patients and spouses considered “a good marriage” to be essential for coping with PD (Carter and Carter, 1994) and couples coping with stroke reported drawing on existing relationship strengths to cope with the changes associated with the stroke (McCarthy and Bauer, 2015). The compatibility of preexisting communication and coping styles also favored positive dyadic coping in MS couples. “In-sync” couples from the sample of Starks et al. (2010), i.e., couples who had adjusted well to living with MS, were often characterized by compatible world views and communication styles. In contrast, Boland et al. (2012) reported that some couples who had difficulty adjusting to MS presented with coping styles that had once been complimentary, but became oppositional in the face of added stress due to the health problem. That is, while differing coping styles were functional pre-impairment because they complemented each other well, these differences went on to cause tension and friction between partners once MS generated more stress for the couple.

A second factor that seemed to favor positive dyadic coping was sensitivity between partners. Being attuned to each other's feelings and needs helped couples cope with locomotor disability (Schembri Lia and Abela, 2019), and mutual understanding and patience were central to coping with SCI (Chan, 2000). Noticing one's own negative behaviors toward the partner and actively engaging to counteract them, e.g., by countering negative comments with expressions of affection, was an indicator of sensitivity reported in couples coping with one partner's hearing loss (Yorgason et al., 2007). In contrast, communication breakdown in MS couples seemed to occur as a consequence of repeated insensitivities when one partner negated the other's experiences, e.g., by trivializing them or by offering unsolicited positive reevaluation (Wawrziczny et al., 2019). Insensitivities of the partner were also experienced by individuals with SCI. Patients generally reported that they needed more emotional than instrumental support while they perceived their spouses to mainly provide instrumental support (Ghafari et al., 2014).

Thirdly, acceptance of the disability and its consequences also seemed to favor positive dyadic coping. For instance, Smith and Shaw (2017) concluded that PD couples fared well when they assimilated PD into their lives, that is, when couples acknowledged that PD required changes to their lifestyle. This allowed patients to retain more agency and thus provided them with more opportunities to be involved in coping. In contrast, lack of acceptance hindered constructive dyadic coping. Some stroke survivors rejected the role changes within their relationships, particularly their own role as care recipient, thus potentially abstaining from expressing their needs for support (Quinn et al., 2014). Similarly, some couples coping with MS reported they did not want to give much space to the disease, i.e., they were not willing to acknowledge its place within the relationship. Consequently, communication and mutual support between the partners deteriorated over time (Wawrziczny et al., 2019).

Other, less frequently mentioned factors that particularly hindered sharing of personal experiences included fear that one's feelings would get hurt (Jeyathevan et al., 2019) and the perceived unpredictability of the partner's reaction (O'Keeffe et al., 2020).

### Dyadic Adjustment

As depicted in Figure 2, several studies indicated that dyadic challenges and dyadic coping are related to overall dyadic adjustment following disability. Early quantitative findings on couples coping with SCI suggested that most partners (84%) were overall satisfied with their relationship (Kreuter et al., 1994). Marital adjustment and marital satisfaction did not differ between SCI couples and couples with two healthy partners, whereas SCI couples even reported significantly higher marital stability (Yim et al., 1998). Overall marital adjustment in couples coping with PD was not significantly different from population norms. However, when considering subscales, consensus was significantly lower and cohesion was significantly higher in PD couples than in the general population (Carter and Carter, 1994). Data from couples coping with TBI suggested that about half of patients and partners reported clinically significant levels of marital dissatisfaction. However, ratings of marital instability were lower with roughly one quarter of participants reporting their marriage was unstable (Kreutzer et al., 2016). There was evidence for a decline of relationship satisfaction over 1 year for couples coping with MS. However, whether this decline was directly related to coping with MS is difficult to establish given that baseline measurements were taken at a mean number of 15.66 years (SD = 10.62) since onset of symptoms (Samios et al., 2015).

Qualitative studies focused more on the unfolding and often circular process of dyadic challenges and the related stress experience, dyadic coping, and adjustment. They often concluded that couples experienced phases of crisis when stress exceeded available dyadic coping resources and phases of (re-)adjustment when couples were able to balance out stress through coping efforts. Some studies came to the overall conclusion that couples did not adjust well to physical or sensory disability, as is evidenced, for example, by the theme of “the alienated couple” reported by Wawrziczny et al. (2019) in MS couples. Other examples include permanently altered communication in couples coping with stroke (Croteau et al., 2020), or the finding that the majority of spouses of TBI patients felt their love had changed toward a caring relationship lacking romantic aspects (Bodley-Scott and Riley, 2015). Other qualitative studies in this review, however, presented a more balanced account. The relationships of couples coping with TBI were captured by the somewhat opposing themes of “broken bonds” and “new dynamics” (O'Keeffe et al., 2020). Similarly, SCI couples' adjustment post-injury was described as laying on a continuum from “constriction/stagnation” to “connection/flexibility” (Engblom-Deglmann and Hamilton, 2020). Some couples witnessed a deterioration of their relationships, while others were able to maintain or rebuild their relationships (Jeyathevan et al., 2019). Accounts of (intermittent) deterioration of the relationship with subsequent adjustment to varying degrees were most common (Chan, 2000; Dickson et al., 2010; Boland et al., 2012; McCarthy and Bauer, 2015; Martin, 2016; Anderson et al., 2017; Smith and Shaw, 2017; Glade, 2018).

## Discussion

The aims of this review were to identify dyadic challenges due to one partner's chronically disabling physical or sensory health impairment that may strain the couple relationship and to summarize evidence regarding dyadic coping with these challenges. Findings from qualitative and quantitative research were integrated to provide a comprehensive account of available evidence. Thirty-six publications matched the inclusion criteria. The results clearly underline that impairments and their consequences affect both members of the couple and generate we-stress (Bodenmann, 2005). In other words, disability is an interpersonal experience in close relationships. This has repeatedly been found in other chronic health conditions such as cancer (Hagedoorn et al., 2008), diabetes (Lister et al., 2013), and cardiovascular disease (Trump and Mendenhall, 2017). The review also indicated that dyadic challenges were largely comparable across health impairments. Couples experienced similar challenges although they were not coping with the same diagnosis. This supports theoretical work on how couples coping with one partner's health condition are faced with a series of common stressors due to changes in the relationship (Rolland, 1994). The type of health condition may influence the relevance and burden of certain changes, but the factors related to maintaining a balanced relationship remain comparable for all couples.

The most frequent dyadic challenges identified in this review were changes in roles and responsibilities of the partners, altered communication, compromised sexual intimacy, and restricted social participation. Altered communication due to functional impairments was particularly relevant in couples coping with sensory disability, whereas sexual intimacy was most strongly compromised in the context of physical disability. This underlines the relevance of contextual factors to fully understand dyadic coping in the context of impaired health (e.g., Berg and Upchurch, 2007). In accordance with the similarity of dyadic challenges, the current review also showed that adaptive dyadic coping strategies were comparable across health impairments. Adopting a we-perspective and conjoint involvement of both partners in coping were crucial for couples. Partners' willingness and effort to mutually share and listen to each other's personal experiences supported conjoint dyadic coping and were beneficial for dyadic adjustment. In other words, couples coping with chronically disabling physical or sensory impairment of one partner fare best when partners stay connected and remain sensitive to each other's experiences and when they join their forces to counteract the potentially deleterious effects of the impairment on their relationship and well-being (see Figure 2).

### De-Emphasizing the “You” and “Me”

Changes in roles and responsibilities are almost inevitable when one partner in a couple faces chronically impaired health. For example, impairments can cause patients who previously worked for pay to reduce or cease their professional activities. Similarly, the transition from romantic partners to caregiver and care recipient is a common experience for most couples. Despite being common, these changes should not be neglected as they strongly contribute to the experience of chronic stress in both partners. Chronic everyday stress, in turn, can have detrimental effects for individual and relational well-being (Bodenmann, 2005; Randall and Bodenmann, 2009). For instance, forced retirement often means a loss of social status and opportunities for social integration for the patient with potential negative effects on their self-esteem (van der Heide et al., 2013). Partners, on the other hand, may need to step in to avoid financial strain for the couple or family. This increases workload and stress for partners. Furthermore, across different health conditions, partners often report feeling overwhelmed with their new “identity” as caregivers and with caregiving tasks (Kang et al., 2011; Mausbach et al., 2012; McCarthy and Bauer, 2015). Patients, on the other hand, may experience frustration when they become dependent on care provided by their spouse. In particular, overprotection of partners toward patients can threaten patients' sense of autonomy and control. The frustration about their undermined autonomy contributes to the experience of stress in patients and can trigger conflict in the couple (Kuijer et al., 2000; Dalteg et al., 2011).

Beyond generating chronic stress for both partners, the role changes couples experience when coping with chronic health impairments in one partner disturb the delicate balance of autonomy and (inter-)dependence within a couple. Such imbalances occur as a function of ascribing a diagnosis to one partner. This partner is labeled as “the patient” who is normatively expected to be the recipient of care and support. The other partner becomes “the partner” who is expected to provide care and support (Leuchtmann and Bodenmann, 2017). Such a juxtaposition of role expectations may jeopardize the perceived balance of support, e.g., the equity of dyadic coping. Inequity in support transactions can undermine individual and relational well-being. For example, receiving support without reciprocating it was associated with poorer mood in the recipient (Gleason et al., 2003) and inequity of dyadic coping was associated with lower personal health and relationship satisfaction (Gmelch and Bodenmann, 2007; Iafrate et al., 2012). These associations also hold in times of heightened stress. Inequity of dyadic coping was associated with more depressive symptoms in couples shortly after the birth of their first child (Meier et al., 2020), in couples facing a kidney transplantation (Tkachenko et al., 2019), and in patients with a major depressive episode (Meier et al., 2021). Couples thus seem to have a continued need for equitable coping contributions of both partners even when factors such as chronically impaired health of one partner challenge balanced coping efforts.

Couples' continued need for balanced contributions to coping underlines the importance of conjoint dyadic coping efforts. As the results of this review showed, coping together rather than individually is crucial for couples to best adjust to chronically impaired health of one partner (e.g., Starks et al., 2010). This is consistent with the findings that conjoint forms of dyadic coping are strongly related to better individual and dyadic adjustment in couples coping with impaired health (e.g., Traa et al., 2015). However, normative role expectations for patients and partners contradict conjoint and balanced involvement of both partners in dyadic coping. The view that the patient presents with the impairment and needs care and support while the healthy partner provides any care and support the patient may need contributes to a focus on the “you” and “me” in couples and neglects couples' interpersonal experience of disability. De-emphasizing patient and partner roles, instead, allows for a much more nuanced perspective on couples coping with impaired health: Both partners experience suffering related to the consequences of the impairment, but they also both have resources to jointly cope with these consequences (Leuchtmann and Bodenmann, 2017). De-emphasizing patient and partner roles and de-emphasizing the “you” and “me” will help couples focus on their united strength and resilience.

Couples themselves, their immediate social environment as well as healthcare and social service providers can all contribute to de-emphasizing rigid patient and partner roles. Healthcare and social services generally have one client, namely the person with a health impairment, who is assigned medical or other assistance. However, providers can support a dyadic perspective in various ways. For instance, they can address possible impacts of the impairment on the couple relationship in consultation. Studies indicate that this is a commonly expressed need. For instance, in a study on couples coping with stroke included in this review, most participants said they felt that the rehabilitation team should initiate conversations about post-stroke sexuality. However, only 3 of 29 interview participants reported that a physician or psychologist had discussed sexual adjustment with them (Schmitz and Finkelstein, 2010). Similar discrepancies between needs for discussion of sexual adjustment post-diagnosis have been reported in cancer (e.g., Lindau et al., 2011; Sporn et al., 2015). Further options for healthcare staff to de-emphasize patient and partner roles include, among others, explicitly asking the patient to bring their spouse to appointments or discussing the option of referral to couple counseling or psychosocial interventions targeted at couples coping with health impairments (see, e.g., Martire et al., 2010; Badr and Krebs, 2013). The immediate social environment can also contribute to de-emphasizing patient and partner roles. They may, for example, ask about all family members and whether they need support. In a study reviewed here, the wife of a man who had sustained SCI talked about how people in their social circle usually asked only about her husband, leaving herself to feel unrecognized (Dickson et al., 2010). Couples also experienced their social circle gradually diminishing because friends would not know how to openly talk about the injury (Engblom-Deglmann and Hamilton, 2020). Events like these may be reduced if couples' friends and kin are educated about the impairment, how they can talk about its consequences for the couple and how to support the couple. The couple can enhance others' understanding by addressing such topics openly with their social network to increase awareness for their experiences. The partners can further contribute to de-emphasizing their respective roles by mutually inquiring about each other's experiences.

### Strengthening the “We”

When the “you” and “me” are de-emphasized, couples can focus on strengthening the “we.” The results of this review show that chronically disabling health impairments of one partner strongly affect both partners. Consequently, our results highlight that adopting a we-perspective is most beneficial when coping with dyadic challenges related to the impairment (e.g., Freeman et al., 2017). In line with the notions of we-stress and we-disease (Kayser et al., 2007; Bodenmann et al., 2016) and with communal coping theory (Lyons et al., 1998; Helgeson et al., 2018), focusing on the health impairment as “our” problem contributes to good dyadic and individual adjustment. The works of Skerrett (1998, 2003) and Fergus (2011) on couples coping with cancer have shown that viewing cancer as “our problem” is an important source of resilience and promotes optimal functioning of the couple in the face of adversity. Similarly, when couples considered diabetes to be a shared problem and both partners were involved in diabetes management, patients reported better relationship quality and partners reported lower distress (Helgeson et al., 2017). First-person plural noun use (“we-talk”) as a proxy for a we-perspective has been linked to positive health outcomes in patients with heart or lung problems (Rohrbaugh et al., 2012) and heart failure patients (Rohrbaugh et al., 2008). Spouses' higher shared appraisals of diabetes were related to weaker associations between patients' self-efficacy and distress. In other words, patients with low self-efficacy were buffered against poor adjustment when their spouses considered diabetes a shared problem (Zajdel et al., 2018).

As adopting a we-perspective to coping with chronic health impairments is clearly beneficial for couples, investigating how such a we-perspective develops is crucial. Findings from the current review suggest that partners' mutual sharing of their personal experiences may be one factor that contributes to developing a we-perspective (e.g., Anderson et al., 2017). Previous research shows beneficial effects of mutually sharing personal experiences for relational functioning in general. In daily diaries of healthy couples, self-disclosure and partner disclosure contributed to same-day perceived intimacy (Laurenceau et al., 2005). In breast cancer patients and their cohabiting partners, mutual expression and discussion of feelings related to cancer around the time of surgery was associated with greater relationship satisfaction 9 months later (Manne et al., 2006). Positive associations between mutual constructive communication and relationship functioning were confirmed in a systematic review on couples coping with cancer (Traa et al., 2015). One explanation for the high relevance of mutual sharing may be the couples' need to negotiate cognitive representations of the health impairment. These representations shape their approach to coping—more individual vs. more dyadic coping. The representations can, however, not be expected to be congruent between partners given, for example, that the patient directly experiences symptoms while the partner only has indirect access to experiences related to the impairment. Thus, mutually sharing their personal experiences helps both partners align their respective cognitive representations of the health impairment more closely so that they can jointly develop the most effective approaches to coping (Badr and Acitelli, 2017). However, future research is needed to gain more insight into the cognitive and communicative processes involved in developing couples' we-perspectives when coping with impaired health.

Furthermore, future research should focus on holding back from sharing personal experiences related to impaired health. Findings from this review suggest that holding back from sharing can undermine a we-perspective by increasing emotional distance between partners. This is consistent with the assumption that holding back is relationship-compromising (Manne and Badr, 2008) which is, for instance, supported by a negative association of holding back with relationship intimacy in couples coping with prostate cancer (Manne et al., 2015). Protective buffering, i.e., efforts to hide or deny concerns from one's partner (Coyne and Smith, 1991), also seemed to have adverse psychosocial effects in couples coping with cancer. The more participants buffered their partners and the more they felt buffered by their partner, the lower their relationship satisfaction (Langer et al., 2009). Data from ecological momentary assessment in cancer couples' daily lives confirmed the negative association between holding back and one's own relationship satisfaction. They also suggested interpersonal effects, i.e., holding back was negatively associated with one's partner's relationship satisfaction (Langer et al., 2018). Protective buffering also had negative effects on intimacy in cancer couples (Perndorfer et al., 2019). These findings support the assumption that holding back from sharing personal experiences may signal distancing of the partners and thus erode couples' sense of being a unit. However, future research is needed to disentangle the differential contributions of mutual sharing and holding back to developing a we-perspective. Furthermore, investigating conditions that favor mutual sharing and reasons for holding back will foster our understanding of the we-perspective.

In sum, strengthening the “we” in couples coping with chronic health impairments contributes to dyadic adjustment by focusing the couples' attention on shared coping resources. This can be achieved when both partners reciprocally share their experiences, concerns, and needs generating a narrative of being “in it together.”

### Strengths and Limitations

This review adds to our understanding of disability as an interpersonal experience. It represents an important step to identifying similarities and differences in dyadic coping and dyadic adjustment across different health impairments with varying contextual factors such as disease progression that have been rather neglected in dyadic coping research. The review considers qualitative and quantitative studies to ensure a comprehensive synthesis of available evidence and comparison of findings across research designs. This allows to check more in depth for the robustness of findings. In this review, qualitative studies were particularly helpful to explore changes in the couple relationship in detail. They also captured the temporal unfolding of dyadic coping as a prolonged process. In contrast, quantitative studies helped to frame the significance of identified dyadic challenges and coping elements by indicating how frequent and pronounced these phenomena were. The findings clearly suggest that stressors for couples are comparable across chronically disabling health impairments as are dyadic coping strategies that foster good dyadic adjustment despite chronic stress. This can inform the development of psychosocial interventions which aim to enhance couple relationships strained by impaired health.

Nonetheless, there are several limitations to this systematic review. First, although cognitive impairments and their impact on the couple relationship were not the focus of this review, some of the reviewed studies may have included patients who presented with cognitive impairments. Cognitive impairments pose specific challenges for couples. For instance, in dementia, relationship functioning seems to be strongly related to behavioral problems of the patient (Quinn et al., 2009). However, except for one study that focused on stroke-related aphasia and its impact on couples' communication (Croteau et al., 2020) and two studies suggesting that personality changes in TBI patients might have contributed to emotional distance between the partners (Bodley-Scott and Riley, 2015; O'Keeffe et al., 2020), no studies showed indications that cognitive impairments were responsible for the relationship challenges summarized in this review. Second, the current review focused on dyadic processes and their relation to dyadic outcomes. As such, studies investigating individual variables (e.g., illness perceptions, depressive symptoms) in relation to dyadic variables (e.g., relationship satisfaction, dyadic coping) were excluded. Such studies make a unique contribution to our understanding of the relevance of couple relationships for individual well-being. In the current review, however, the unit of analysis is the couple and the emphasis lies on the interdependence of both partners' cognitions, emotions, and actions. Third, the focus on dyadic processes and outcomes may have favored the inclusion of qualitative over quantitative research. The semistructured interview is the most common method of qualitative data collection. It offers researchers the opportunity to jointly interview partners, thus creating a setting that fosters exchange on dyadic experiences. The quantitative questionnaire, in contrast, requires respondents to answer separately and is thus more prone to capturing individual experiences. However, most of the included qualitative studies separately interviewed partners and did thus not benefit from the potential advantages of the dyadic setting. Fourth, we included studies with samples consisting of spousal caregivers and other (family) caregivers. To ensure findings were not confounded with stressors relevant to other forms of close relationships than the spousal/romantic type, we only extracted results that were clearly attributable to romantic relationships, either based on topic (e.g., sexual activity) or respondent (e.g., quotes from spouses vs. parents of patients). Fifth, for the majority of the studies included in this review, we identified concerns about methodological quality. Due to the novel approach of integrating evidence across research designs, we did not limit reporting of results to studies with high-quality ratings. Conclusions drawn from the review's findings should thus be appraised with caution. The identified quality concerns show that the field is in need of continued high-quality research efforts. It will particularly benefit from studies, quantitative and qualitative, taking into consideration the development of the discussed processes over time. For instance, studies with cohorts of couples with varying time since onset of symptoms can give more insight into developmental phases in dyadic coping with impaired health. Longitudinal studies in which couples report on dyadic challenges, dyadic coping, and adjustment across several time points can further add to the existing evidence.

### Suggestions for Future Research

The interpersonal experience of disability in close relationships is an innovative area of research that will greatly profit from intensified research efforts. For instance, in line with the above rationale for more cohort and longitudinal studies on couples coping with disabling health impairments, further research is needed to identify factors that contribute to the development or erosion of a we-perspective in couples coping with chronically impaired health. Investigating the differential contributions of mutually sharing and holding back from sharing personal experiences is one avenue for future research. The findings from this review further suggest that substantial changes in couples' sexual relationships may undermine partners' emotional connectedness. Feeling increasingly disconnected from one's partner may gradually erode a previously established we-perspective. The complex relationship between sexual intimacy and dyadic adjustment in the case of chronic health impairments should thus be investigated more in depth. Similarly, couples in this review often reported restrictions in social participation. This contributed to a lack of shared enjoyment that may also jeopardize closeness and a sense of we-ness. Participation restrictions are often related to insufficient accessibility of public or private spaces. Improving accessibility can thus greatly reinforce couples' opportunities for shared leisure time experiences that strengthen their we-perspective. The effects of social and health policy on couple relationships thus warrant further investigation. Lastly, although a we-perspective is generally beneficial in coping with chronic health impairments, future research should consider cases where a we-perspective may need to be de-emphasized, e.g., in terminal illness. Also, as equity is important in support transactions, congruence or incongruence between partners' we-perspectives and how they relate to dyadic coping can be investigated.

### Practical Implications

The results of this review provide important directions for clinicians who aim to foster couples' coping with chronically disabling health impairments. Most importantly, they suggest that the individual-centered view in standard biomedical care should be paralleled with an interpersonal view of health impairments and disability (Leuchtmann and Bodenmann, 2017). De-emphasizing the roles of patient and partner is in line with couples' perceptions of going through treatment together (e.g., Freeman et al., 2017). Involving both partners in interventions acknowledges this interpersonal experience and shows better efficacy than individual care. For example, in interventions to remedy the psychosocial effects of chronic illness, involving both partners was more beneficial than standard medical care and psychosocial interventions for partners only (Martire et al., 2004, 2010). Viewing the couple as the target of an intervention contributes to de-emphasizing patient and partner roles and practitioners can build on relationship-enhancement interventions for community samples, e.g., the Couples Coping Enhancement Training (CCET; Bodenmann and Shantinath, 2004). The central elements of CCET are communication and conflict resolution, psychoeducation about the deleterious effects of stress, and practical training of dyadic coping skills. Fostering open communication and partners' conjoint dyadic coping efforts resonates with the general importance of strengthening the “we” in couples coping with chronic health impairments. CCET has been adapted for use in couples coping with breast or gynecological cancer (Heinrichs and Zimmermann, 2007), and it has proven to be effective at improving individual well-being and dyadic skills to cope with cancer.

However, findings from this review also suggest some specificities of chronically disabling physical and sensory impairments that should be considered for optimal care: Firstly, some impairments alter communicative abilities of patients. In order to mitigate potential aversive consequences for couple communication, couples need information on specific treatment options such as speech-language therapy or audiological rehabilitation. Secondly, physical impairments and symptoms such as fatigue may interfere with sexual function and sexual activity across a wide variety of health conditions. Healthcare providers should thus actively discuss sexual intimacy with couples and address ways to deal with such changes. Thirdly, couples often experience restrictions in social participation. They should thus be empowered to openly address such issues, for example, in the family or social circle. Additionally, improving accessibility of public spaces can greatly improve couples' opportunities for social participation, underlining the role of public policy for individual and community health. Overall, interdisciplinary networking seems to be crucial to foster optimal adjustment to chronically disabling conditions beyond the individual patient.

In general, when interacting with couples facing health impairments, professionals across disciplines should be vigilant to detect indications of stressful dyadic changes such as sudden role changes or reduced opportunities for social participation. Conversely, they may also want to validate beneficial, relationship-enhancing behaviors. For example, they may praise partners who share their feelings and struggles with regard to their partner's impairment rather than discourage such sharing by exclusively focusing on the person with the impairment. Professionals may also encourage couples to share their experience not only with each other but with their friends and kin as well. Couples expressing apprehensions that sharing personal experiences, especially negative ones, may hurt or burden others may be informed about research pointing to the contrary. Barriers to sharing and open communication may be countered with various types of supportive interventions, e.g., communication or social competence training, self-help groups, or online communities. All these measures require professionals to develop their own sensitivity with regard to the interpersonal dimension of impaired health. Consistently integrating elements of systemic thinking into professional training in healthcare and beyond is thus crucial.

Finally, while we strongly urge to de-emphasize the roles of patient and partner in the healthcare system, their partly differing experiences are undeniable and should not be negated. Instead, the partners need reassurance that temporal shifts and imbalances between partners are inevitable (e.g., Rolland, 1994), but that they also have the ability to renegotiate roles and responsibilities within their relationship. This may empower couples to overcome times when the stress related to coping with chronically impaired health feels overwhelming.

## Conclusions

In close relationships, disability is a profoundly interpersonal experience. Dyadic challenges due to disability are manifold and they are comparable across different underlying impairments. If couples do not exert the necessary dyadic coping, changes in roles and responsibilities, communication, sexual intimacy, and social participation can lead to deterioration of the relationship. Couples cope best when they adopt a we-perspective, that is, when they engage in open communication about both partners' experiences and when they join their forces to develop new outlooks for the relationship. De-emphasizing the roles of patient and partner in favor of viewing both partners as resourceful contributors to each other's well-being thus strengthens the couple as a unit.

## Data Availability Statement

The original contributions generated for the study are included in the article/Supplementary Material, further inquiries can be directed to the corresponding author/s.

## Author Contributions

ICB and GB conceptualized the review. ICB conducted the systematic literature search, assessed article eligibility, extracted data, structured the results, and wrote the first draft of the manuscript. ICB, FM, and GB revised the manuscript. GB supervised the review. All authors have read and approved the final version of the manuscript for submission.

## Conflict of Interest

The authors declare that the research was conducted in the absence of any commercial or financial relationships that could be construed as a potential conflict of interest.
