# Supplementary material for: Disability as an Interpersonal Experience: A Systematic Review on Dyadic Challenges and Dyadic Coping When One Partner Has a Chronic Physical or Sensory Impairment
Source: Front Psychol. 2021 Mar 1;12:624609. doi: 10.3389/fpsyg.2021.624609 (PMC7959177; doi:10.3389/fpsyg.2021.624609)
Supplement: Supplementary file 1 [file Data_Sheet_1.docx]

Supplementary Material:

Complete Search Strings for EBSCOhost Search

# Studies on Dyadic Challenges

(TI(couple* OR Dyad* OR spous* OR “significant other*” OR marital OR marriage OR “committed relationship*”) OR AB(couple* OR Dyad* OR spous* OR “significant other*” OR marital OR marriage OR “committed relationship*”)) AND (TI(adjustment OR “relationship satisfaction” OR “relationship quality”) OR AB(adjustment OR “relationship satisfaction” OR “relationship quality”)) AND (TI(disab* OR handicap*) OR AB(disab* OR handicap*))

(TI(couple* OR Dyad* OR spous* OR “significant other*” OR marital OR marriage OR “committed relationship*”) OR AB(couple* OR Dyad* OR spous* OR “significant other*” OR marital OR marriage OR “committed relationship*”)) AND (TI(adjustment OR “relationship satisfaction” OR “relationship quality”) OR AB(adjustment OR “relationship satisfaction” OR “relationship quality”)) AND (TI("sensory loss" OR "sensory impairment" OR "sensory dysfunction" OR "vision loss" OR "visual impairment" OR "visually impaired" OR "vision impairment" OR "low vision" OR Blind OR blindness OR "hearing loss" OR "hearing impairment" OR "hearing impaired" OR "hard of hearing" OR Deaf OR deafness OR "dual sensory loss" OR Deafblind* OR “vision disorder*” OR “eye disorder*” OR “hearing disorder*”) OR AB("sensory loss" OR "sensory impairment" OR "sensory dysfunction" OR "vision loss" OR "visual impairment" OR "visually impaired" OR "vision impairment" OR "low vision" OR Blind OR blindness OR "hearing loss" OR "hearing impairment" OR "hearing impaired" OR "hard of hearing" OR Deaf OR deafness OR "dual sensory loss" OR Deafblind* OR “vision disorder*” OR “eye disorder*” OR “hearing disorder*”))

(TI("dyadic coping" OR "communal coping" OR "collaborative coping" OR "coping congruence" OR "cooperative coping" OR “couple coping” OR "relationship-focused coping" OR “spousal support” OR “partner social support”) OR AB("dyadic coping" OR "communal coping" OR "collaborative coping" OR "coping congruence" OR "cooperative coping" OR “couple coping” OR "relationship-focused coping" OR “spousal support” OR “partner social support”)) AND (TI(disab* OR handicap* OR impairment*) OR AB(disab* OR handicap* OR impairment*))

# Studies on Dyadic Coping

(TI("dyadic coping" OR "communal coping" OR "collaborative coping" OR "coping congruence" OR "cooperative coping" OR “couple coping” OR "relationship-focused coping" OR “spousal support” OR “partner social support”) OR AB("dyadic coping" OR "communal coping" OR "collaborative coping" OR "coping congruence" OR "cooperative coping" OR “couple coping” OR "relationship-focused coping" OR “spousal support” OR “partner social support”)) AND (TI(disab* OR handicap* OR impairment*) OR AB(disab* OR handicap* OR impairment*))

(TI("dyadic coping" OR "communal coping" OR "collaborative coping" OR "coping congruence" OR "cooperative coping" OR “couple coping” OR "relationship-focused coping" OR “spousal support” OR “partner social support”) OR AB("dyadic coping" OR "communal coping" OR "collaborative coping" OR "coping congruence" OR "cooperative coping" OR “couple coping” OR "relationship-focused coping" OR “spousal support” OR “partner social support”)) AND (TI("sensory loss" OR "sensory impairment" OR "sensory dysfunction" OR "vision loss" OR "visual impairment" OR "visually impaired" OR "vision impairment" OR "low vision" OR Blind OR blindness OR "hearing loss" OR "hearing impairment" OR "hearing impaired" OR "hard of hearing" OR Deaf OR deafness OR "dual sensory loss" OR Deafblind* OR “vision disorder*” OR “eye disorder*” OR “hearing disorder*”) OR AB("sensory loss" OR "sensory impairment" OR "sensory dysfunction" OR "vision loss" OR "visual impairment" OR "visually impaired" OR "vision impairment" OR "low vision" OR Blind OR blindness OR "hearing loss" OR "hearing impairment" OR "hearing impaired" OR "hard of hearing" OR Deaf OR deafness OR "dual sensory loss" OR Deafblind* OR “vision disorder*” OR “eye disorder*” OR “hearing disorder*”))

(TI("dyadic coping" OR "communal coping" OR "collaborative coping" OR "coping congruence" OR "cooperative coping" OR “couple coping” OR "relationship-focused coping" OR “spousal support” OR “partner social support”) OR AB("dyadic coping" OR "communal coping" OR "collaborative coping" OR "coping congruence" OR "cooperative coping" OR “couple coping” OR "relationship-focused coping" OR “spousal support” OR “partner social support”)) AND (TI("spinal cord injury" OR Paraplegia OR Tetraplegia OR Hemiparesis OR hemiplegia OR "traumatic brain injury" OR "multiple sclerosis" OR arthritis OR parkinson OR "parkinson's disease" OR stroke) OR AB("spinal cord injury" OR Paraplegia OR Tetraplegia OR Hemiparesis OR hemiplegia OR "traumatic brain injury" OR "multiple sclerosis" OR arthritis OR parkinson OR "parkinson's disease" OR stroke))
